# Computational Design of Macrocyclic Binders of S100B(ββ): Novel Peptide Theranostics

**DOI:** 10.3390/molecules26030721

**Published:** 2021-01-30

**Authors:** Srinivasaraghavan Kannan, Pietro G. A. Aronica, Thanh Binh Nguyen, Jianguo Li, Chandra S. Verma

**Affiliations:** 1 Bioinformatics Institute, Agency for Science, Technology and Research (A*STAR), 30 Biopolis Street, #07-01 Matrix, Singapore 138671, Singapore; pietroa@bii.a-star.edu.sg (P.G.A.A.); nguyenbinhchem@gmail.com (T.B.N.); lijg@bii.a-star.edu.sg (J.L.); 2 Singapore Eye Research Institute, Singapore 169856, Singapore; 3 School of Biological Sciences, Nanyang Technological University, 60 Nanyang Drive, Singapore 637551, Singapore; 4 Department of Biological Sciences, National University of Singapore, 14 Science Drive 4, Singapore 117543, Singapore

**Keywords:** S100B, molecular dynamics, peptide design, stapled peptides, PPI

## Abstract

S100B(ββ) proteins are a family of multifunctional proteins that are present in several tissues and regulate a wide variety of cellular processes. Their altered expression levels have been associated with several human diseases, such as cancer, inflammatory disorders and neurodegenerative conditions, and hence are of interest as a therapeutic target and a biomarker. Small molecule inhibitors of S100B(ββ) have achieved limited success. Guided by the wealth of available experimental structures of S100B(ββ) in complex with diverse peptides from various protein interacting partners, we combine comparative structural analysis and molecular dynamics simulations to design a series of peptides and their analogues (stapled) as S100B(ββ) binders. The stapled peptides were subject to in silico mutagenesis experiments, resulting in optimized analogues that are predicted to bind to S100B(ββ) with high affinity, and were also modified with imaging agents to serve as diagnostic tools. These stapled peptides can serve as theranostics, which can be used to not only diagnose the levels of S100B(ββ) but also to disrupt the interactions of S100B(ββ) with partner proteins which drive disease progression, thus serving as novel therapeutics.

## 1. Introduction

**Figure 1:**
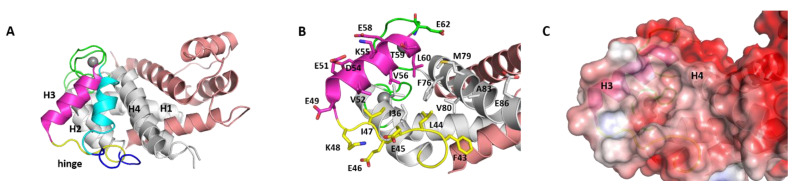
Structures of S100B(ββ). (**A**) Cartoon representation of superimposed apo and calcium bound conformations of the S100B(ββ) protein. Each monomer in the dimer is coloured separately (monomer 1: grey, monomer2: salmon) with H2 and hinge in monomer 1 in its apo (cyan, blue) and calcium bound (magenta, yellow) states, hinge regions (green) and bound calcium (grey sphere) are highlighted. (**B**) The residues from the hydrophobic pocket are highlighted in sticks with colouring similar to A. (**C**) Electrostatics surface representation (red to blue colours represent electrostatic potentials ranging from −5 to +5 kcal/mol) of the calcium bound S100B(ββ) protein dimer; the grey/white colour is localized to the hydrophobic pocket.

S100B(ββ) is among the most extensively studied members of a highly conserved group of Ca^2+^-binding proteins engaged in multiple protein–protein interactions (PPI) [1,2,3,4,5]. Its functional unit is a dimer (homo or hetero) which can be disulfide-linked at the dimer interface or exist as a noncovalent dimer under reducing conditions. In the reduced form, the two subunits of S100B(ββ) associate tightly (Kd < 500 pM) into a compact dimer which is characterized by a highly charged surface and an extensive hydrophobic interface [6,7,8,9,10,11,12,13,14]. Each subunit of S100B(ββ) contains four alpha helices namely (helices h1, h2, h3 and h4) with two loops (loop1 and loop2) connecting h1 to h2 and h3 to h4, respectively. The first helix–loop–helix motif connecting h1, loop1, h2 is referred to as a pseudo-EF-hand, whereas the second helix–loop–helix motif connecting h3, loop2, h4 makes up the canonical EF-hand. These two motifs are connected by a “hinge” region, which consists of 10–12 residues and is very important for target interactions. Binding of Ca^2+^ results in a large conformational change in S100B, with h3 oriented perpendicular to h4, exposing a hydrophobic cleft made up of residues from h2, h3 and h4, and is the region where molecular targets bind (Figure 1). High levels of Ca^2+^ can induce the formation of S100B(ββ) tetramers, hexamers and higher order oligomers [15]. Intracellular S100B(ββ) plays a role in the regulation of events such as enzyme activity and protein phosphorylation [16,17,18], while secreted S100B(ββ) is associated with paracrine, autocrine and endocrine properties [16,17,18].

S100B(ββ) is a clinically validated marker for malignant melanoma (elevated only in tumours) [19,20] and traumatic brain injury (TBI) [21,22,23] and has been associated with cancer progression in multiple myeloma (MM), NSCLC/SCLC, pancreatic cancer, etc., making it a potential target [24,25,26,27,28]. It is known to target p53 oligomerization and DNA binding [29,30,31,32,33], reducing their levels in multiple myeloma and in glioma cells [34,35]. Elevated levels of S100B(ββ) also dysregulate ERK/RSK signalling and increase cell survival in malignant melanoma [36,37]. In addition, S100B(ββ) proteins interact with p53, p21 and receptor for advanced glycation end-products (RAGE), contributing to the degradation of the extracellular matrix resulting in metastasis [38].

The therapeutic targeting of S100B(ββ) with small-molecule inhibitors is being pursued for the treatment of cancers, particularly MM. This has resulted in a wealth of structural and biochemical data on novel small molecules disrupting the S100B(ββ)-p53 interactions, although cellular activity has not yet been validated. Success in the development of small molecules targeting protein–protein interactions has been limited [39,40,41,42]; these stem from the large and usually shallow nature of PPIs interfaces. Recently, there has been great enthusiasm for using peptides and peptidomimetics to interrupt PPIs, opening the gates for accessing a vast landscape of intracellular processes that were traditionally deemed undruggable [43,44]. The peptides have been derived from the interfacial region of one of the partners in the PPI, and hence, their derivatives have demonstrated specificity and indeed high affinity upon optimization [45,46,47], together with high biocompatibility and low toxicity. Several peptides and peptidomimetic candidates are currently in clinical trials targeting several diseases, and more than 50 have been approved by the FDA. Despite these advances, peptide developments have generally suffered from proteolytic sensitivity and cell permeability. These challenges are slowly being addressed with advances in macrocyclization and chemical modifications [45,46,47,48,49]. Macrocyclization (a) constrains the peptides into their bound conformations, thus resulting in reduced entropic penalties normally incurred when the normally unconstrained peptides bind to their targets; (b) results in opportunities for attenuating off-target effects resulting from the increased specificity; (c) combined with modifications in backbone and/or side-chains can confer proteolytic resistance; (d) can enhance cell permeability largely by improved stability of the intramolecular hydrogen bonds that enable permeabilization across apolar cell membranes. One of the techniques for macrocyclization that has been gaining popularity is that of stapling, whereby olefin cross-links are used to bridge unnatural amino acids (that have been strategically introduced along the peptide chain) carrying alpha-methyl alkenyl side chains [48,49,50,51]; this is especially effective if the structure to be constrained is helical. The staples are located so that they do not interfere with binding, can be of different lengths, with the most common ones bridging amino acids across i,i+3 i,i+4, i,i+7 and i,i+11 residues; recently, there have been reports of the successful stapling of non-helical peptides [52]. There are several pathways that have been successfully targeted with stapled peptides [51,53].

We report here a computational study of the design of a set of stapled peptides targeting S100B(ββ), guided by a wealth of experimental data available. Rustandi et al. [54] used NMR spectroscopy to characterize the interaction of S100B(ββ) with a p53 peptide. A 22-residue peptide derived from the C-terminus of p53 (residues 367–388) was observed to bind to the hydrophobic cleft on the surface of S100B(ββ). Bhattacharya et al. [55] reported the solution structure of S100B(ββ) bound to a peptide fragment from the NDR kinase; this peptide binds at the hydrophobic cleft on the surface of S100B(ββ). Dimlich and co-workers [56] identified a 12-mer peptide (TRTK-12) from a bacteriophage random peptide display library screen that bound to the hydrophobic cleft of S100B(ββ) [57]. Jensen et al. [58] reported the crystal structure of a 15-amino-acid synthetic peptide (corresponding to residues 54–68 of RAGE) also bound to the hydrophobic cleft on the surface of S100B(ββ). In all these cases, the S100B(ββ) dimer was bound to two peptide molecules. Gogl et al. [59] determined the structure of several peptide fragments from Ribosomal protein S6 kinase alpha-1 (RSK1) bound to the hydrophobic cleft of S100B(ββ); the peptides adopted diverse binding modes. Uniquely, in this case, only one monomer was occupied by the peptides.

## 2. Comparative Analysis of S100B(ββ)-Peptide Complexes

**Figure 2:**
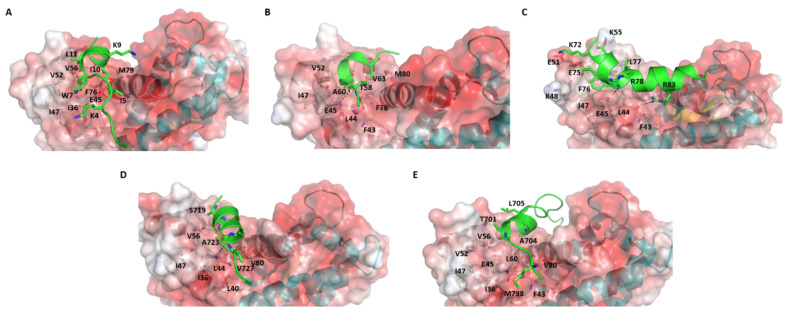
Structures of S100B(ββ)-peptide complexes. Structures of S100B(ββ)—(**A**) TRTK12, (**B**) RAGE, (**C**) NDR, (**D**) RSK_PEP2 and (**E**) RSK_PEP1 complexes are shown. The S100B(ββ) protein dimer is shown as an electrostatic surface (red to blue colours represent electrostatic potentials ranging from −5 to +5 kcal/mol) with the two monomers coloured separately (grey, cyan). The bound peptide is shown as cartoon (green) and peptide-protein interacting residues and h-bond interactions are highlighted in sticks and dashed lines, respectively.

We now explore in detail the interactions between the structurally determined S100B(ββ)-peptide complexes to elaborate on the complex landscape of these interactions (Figure 2). TRTK12 (_1_TRTKIDWNKILS_12_) is a synthetic peptide that has been shown to bind as a random coil to S100B(ββ) with the highest binding affinity (*K_d_*~260 nM) of any molecule targeting S100B(ββ); this peptide was found to be part of the C-terminal region (residues 265-276) of actin capping protein CapZ [56]. The solution structure of the Ca^2+^-human S100B(ββ)-TRTK12 complex shows that each S100B(ββ) monomer binds one TRTK12 molecule. The peptide binds at the hydrophobic cleft formed between h3 and h4 mainly through hydrophobic interactions (Figure S1). Peptide residues I5, W7, I10 and L11 interact with S100B(ββ) residues V52, V56, T59 and L60 from helix 3 and F76, M79, V80, A83 and C84 from helix 4. Residue W7 of the peptide is the anchoring residue buried in a hydrophobic pocket formed by residues V56, T59, F76 and V80 of S100B(ββ) (Figure S1). In addition, electrostatic interactions that contribute to the binding of the peptide include a salt bridge between R2 from the peptide and E86 from S100B(ββ) (Figure S1A).

Surprisingly, the solution structure of the same 12 amino acid peptide (TRTK12) bound to rat S100B(ββ) protein shows that the peptide binds to the same hydrophobic region between h3 and h4, and yet adopts a helical conformation (amphipathic helix from residues W7 to S12) with residues 1-6 disordered (Figure 2A) [60]. This is surprising since the two proteins differ only by two amino acids. Even more surprising was the observation that the orientation of the bound peptide is perpendicular to the conformation adopted by this peptide when bound to human S100B(ββ); the N-terminal region of the peptide interacts with the helix h4 in the former and with the hinge region in the latter. The side chain of W7 of the peptide is buried into the hydrophobic pocket formed by residues I36, I47, V52, V56, F76 and M79 (as in the case of the human S100B(ββ) protein) and anchors the peptide into the binding pocket. The binding of the peptide is also largely mediated by hydrophobic residues from S100B(ββ) (residues V52, I47, I36, V 56, F76, M79 and V80) and TRTK12 (I5, W7, I10 and L11). In addition, two electrostatic interactions were observed: (i) K4 from the peptide is close to H42 and E46 from S100B(ββ); (ii) K9 from the peptide is close to E86 from S100B (Figure 2A).

Jensen et al. [58] reported a crystal structure of human S100B(ββ) bound to a 15-amino-acid synthetic peptide that corresponds to residues 54–68 of RAGE (_54_NTGRTEAWKVLSPQG_68_). The RAGE peptide also binds in the hydrophobic pocket formed by helices h3 and h4 on the surface of S100B(ββ). The bound peptide adopts a single helical turn in the middle (from residues 60 to 63), while the flanking residues adopt extended coil structures (Figure 2B). Hydrophobic interactions dominate the binding of the RAGE peptide with the S100B(ββ) protein. The side chains of A60 and V63 from RAGE occupy the hydrophobic pocket made by residues L45, I48, V53, V57, L61, P77 and M80 from S100B(ββ). In addition, the amide and carbonyl backbone of T58 from RAGE form h-bonds with the carbonyl backbone of F44 and the amide backbone of E46 from S100B(ββ). Interestingly, the RAGE peptide also has a tryptophan (W61), but it is exposed to solvent and the observation that the RAGE^W61A^ mutant peptide binds to S100B(ββ) with an affinity similar to the RAGE^WT^ peptide demonstrates that W61 is not critical [58].

Bhattacharya S et al. [55] reported the solution structure of bovine S100B(ββ) bound to a 25 amino acid peptide (_62_KRLRRSAHARKETEFLRLKRTRLGLE_87_) that corresponds to the region between the N-terminal regulatory and catalytic domains of the NDR kinase. Similar to the other S100B(ββ)-peptide complexes, one NDR peptide is found to associate with each subunit of the S100B(ββ) dimer and bound at the hydrophobic cleft between the hinge region and helices h3 and h4. However, unlike the other peptides, the NDR peptide is more aligned to the hinge region of S100B(ββ) rather than to helices h3 or h4 and has very minimal interactions with helix h3 (only at the N-terminal of helix h3) and helix h4 (only at the C-terminal of helix h4). The bound NDR peptide is unstructured at its N-terminus (residues 62−71) and adopts a helical conformation from K72 to L86 (Figure 2C and Figure S1B). The complex is stabilized by both hydrophobic and electrostatic interactions. The side chain of F76 of the NDR peptide is buried into a hydrophobic pocket formed by residues I47, V52 and V56 of S100B(ββ). The NDR peptide residue L77 is involved in hydrophobic interactions with V56, V52 and L44 of S100B(ββ). In addition, a significant number of electrostatic interactions were observed between S100B(ββ) and the NDR peptide. The side chain of peptide residue K72 interacts with the side chain of E49 from S100B(ββ), the side chain of peptide residue E73 interacts with the side chain of K55 from S100B(ββ), the side chain of peptide residue E75 is in close proximity to the side chain of K48 from S100B(ββ), the side chain of peptide residue R78 interacts with the side chain of E45 from S100B(ββ) and the side chain of peptide residue R83 interacts with the backbone of F43 from S100B(ββ).

Recently, Gogl et al. [59] determined the structure of several peptide fragments from RSK1 C-terminal CaMK-type domain (RSK1CTKD) bound to human S100B(ββ). Similar to the other S100B-peptide complex structures, the peptide fragments from RSK1 were also found to be bound at the hydrophobic cleft between the helices h3 and h4 on the surface of S100B. However, unlike the other S100B(ββ)-peptide complexes, here, a single peptide fragment was bound with one S100B(ββ) dimer. It appears that RSK1 can adopt multiple conformational states upon binding to S100B(ββ). In two complexes (RSK1_L_e, RSK1_S_e), the N-terminal region (from residues 696 to 703 referred as RSK1_L_e) and the C-terminal region (from residues 725 to 731 referred as RSK1_S_e) adopt extended coil structures, bound to the hydrophobic clefts (Figure S1C). In model 3, the N-terminal region of RSK1CTKD (RSK1_L_e) is bound to the hydrophobic pocket in one monomer and adopts an extended coil, while the same hydrophobic pocket from the other monomer is occupied by the C-terminal region of RSK1CTKD (from residues 718 to 731 referred as RSK_PEP1), but the peptide adopts a helical structure (from residues 718 to 727) (Figure 2D). In model 4, the RSK1CTKD peptide fragment (residues 697 to 715 referred as RSK_PEP2) is bound to the hydrophobic cleft of one monomer, while the hydrophobic cleft in the other monomer is unoccupied. The middle region of the peptide (residues 701 to 712) adopts a helical structure with the flanking residues disordered (Figure 2E). In the models in which the RSK1CTKD peptide RSK1_L_e adopts extended coil structures, few hydrophobic and polar interactions were observed: the side chains of residues R725 and R726 are close to the side chains of E86 and E45 from S100B(ββ) (Figure S1C). In the models in which the RSK1CTKD peptides RSK_PEP1 and RSK_PEP2 adopt alpha helical structures, only the side chain of Alanine (A704 in RSK_PEP1 and A723 in RSK_PEP2) was found buried into the hydrophobic pocket formed by residues L44, L47, V52, I36, V56, L60 and F76 of S100B(ββ) (Figure 2D,E). In the case of RSK_PEP1, the side chains of peptide residues A699 and T701 occupy the hydrophobic pocket, and a hydrogen bond was observed between the amide backbone of A699 from the peptide and the carbonyl backbone of F43 from S100B (Figure 2E). In the case of RSK_PEP2, the side chains of peptide residues S719, S720 and Q724 occupy the hydrophobic pocket, and a hydrogen bond between the carbonyl backbone of Q724 from peptide with the amide backbone of E45 from S100B(ββ) was observed.

Comparison of these S100B(ββ)-peptide complexes revealed that despite little sequence similarity between the peptides, they all bind to the same hydrophobic pocket on the surface of S100B(ββ), albeit with distinct interactions and diverse binding affinities (from nanomolar to micromolar). It is intriguing that although in the four complexes of S100B(ββ)-peptides (peptides from p53, TRTK12, RAGE and RSK1), the peptides adopt alpha helical conformations and bind at the same pocket (aligning with helices h3 and h4) on the surface of S100B(ββ), yet they all differ from each other in terms of sequence and binding modes. Intriguingly, the same peptides (TRK12 and RSK1) can also adopt completely different conformations (coiled coil structures) yet bind to the same pocket on the surface of S100B(ββ), but in completely different orientations.

## 3. Methods

### Molecular Dynamics (MD) Simulations

Structural models of human S100B(ββ)-peptide complexes were either taken from or modelled based on the available experimental structures. The structure of human S100B(ββ)-NDR was modelled based on the structure of bovine S100B(ββ)-NDR (PDB: 1PSB), while the structure of human S100B(ββ) bound to TRTK12 in an alpha helical conformation was modelled based on the structure of the complex of rat S100B(ββ) bound to TRTK12 in an alpha helical conformation (pdb: 1MWM). The experimental structure of the 3 other complexes, human S100B(ββ) bound to RSK1 (PDB: 5CSN, 5CSF, 5CSJ), human S100B(ββ) bound to TRTK12 (PDB 1MQ1) and human S100B(ββ) bound to RAGE (pdb: 4XYN) were available and used. Simulations were carried out using the protocol taken from our previous work [61]. Each system was subject to MD simulations, which were carried out with the *pemed.CUDA* module of the program Amber18 [62] with the Amber 14SB force field (ff14SB) [63]. Parameterisation of the imaging agents DOTA, NOTA and FAM was carried out using the protocol taken from our previous work [61] with the program NWCHEM [64]. Parameters for staple linkers were used as described in our previous work [65]. For each system, MD simulations were carried out for 100 ns in triplicates. Molecular Mechanics Poisson Boltzmann Surface Area (MMPBSA) methods were used for the calculation of the binding free energies between the peptides and S100B(ββ) [65]. RMSD of the conformations sampled during the simulations with respect to the starting structures (Figure S2) showed that the simulations had stabilized, thus enabling us to use the second half (50ns to 100ns) of the simulations for analysis (the RMSD fluctuations within this region are ~2 Å). In total, 250 conformations were extracted at equal intervals (200 ps) from the last 50 ns of each simulation and were used for the binding energy calculations. Entropy calculations are computationally intensive and do not converge easily and hence are ignored here; hence, the free energies referred to are the enthalpic components of the free energies. All MD simulations and analysis including the binding energy calculations were carried out as discussed in our previous study [61] and are summarized in the supplementary. Simulation trajectories were visualized using VMD [66], and figures were generated using PyMOL [67].

## 4. Results and Discussion

### 4.1. Conformational Dynamics of S100B(ββ)-Peptide Complexes

**Figure 3:**
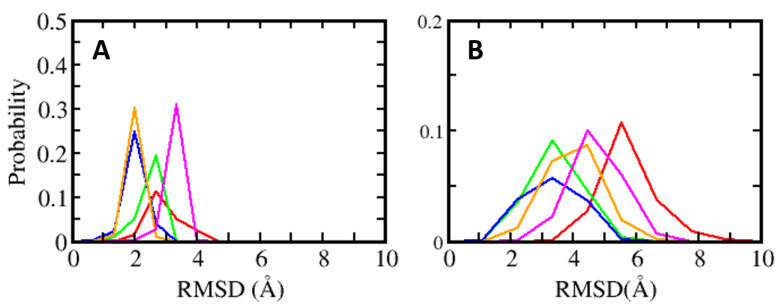
Distributions of root mean square deviation (RMSD) of (**A**) S100B(ββ) (**B**) and the bound peptides (red: NDR, green: RAGE, blue: RSK_PEP1, orange: RSK_PEP2, magenta: TRTK12).

MD simulations were carried out to understand the dynamics of the S100B(ββ)-peptide complexes. In the case of the TRK12 and RSK1 peptides, the simulations were carried out with the peptides in both the extended coil and alpha helical conformations. In the case of the NDR peptides, simulations were carried out with only the alpha helical fragment (residues K72 to L86) as the N-terminal region of the peptide is unstructured and highly flexible, as seen in the NMR ensemble (Figure S1). In the case of the S100B(ββ)-RAGE complex, simulations were carried out with the peptide in an alpha helical conformation. For the simulations, dimeric S100B(ββ) with peptides bound to only one monomer was used. During the molecular dynamics (MD) simulations, the S100B(ββ)-peptide complexes remained stable with no unbinding of peptides observed. The S100B dimer remained stable within RMSD < 4 Å from the experimental structure (Figure 3A). The bound peptides also largely remained stable, but were more flexible than the receptor; the NDR peptide binds in an orientation that is perpendicular to the other peptides (p53, TDTK12, RSK1 and RAGE) and exhibited high flexibility with RMSD ~5.5 Å (Figure 3B). Lack of stable protein–peptide interactions contributes to the increased flexibility of this NDR peptide. The TRTK12 peptide displayed high RMSD of ~4.5 Å (Figure 3B), due to its unstructured N-terminal (residues 1 to 5) region, which remained disordered and exhibited increased flexibility during the MD simulations. Among the other three peptides (RSK_PEP1; RSK_PEP2, which adopt alpha helical conformations, the RAGE peptide, which adopts a single turn helix conformation), exhibited somewhat reduced flexibility with RMSD ~4 Å (Figure 3B). Peptides TRTK12 (TRTK12_e) and RSK1 (RSK1_L_e) are shown to bind to S100B(ββ) in either extended coil or an alpha helical conformations. To investigate what conformation of the peptides is more stable upon binding to S100B, we carried out simulations of the S100B(ββ)-peptide (TRTK12 _e and RSK1_L_e) complexes with the peptides in the extended coil conformation and in the alpha helical conformation. Irrespective of the bound conformation of the peptides, no major difference in the S100B(ββ) protein was observed during the MD simulations (Figure S3A). In contrast, major differences in conformational stability were observed for the peptides bound in extended coil and alpha helical conformations. The RSK1_L_e exhibited RMSD of ~4 Å and ~6 Å for the alpha helical and extended coil conformations, respectively (Figure S3B), while the TRTK12_e peptide did not exhibit any major differences in conformational stability (RMSD of ~4.5 Å) between the alpha helical and extended coil conformations (Figure S3B). Unlike the other S100B(ββ) binding peptides, the TRTK12_e peptide is a high affinity peptide (Kd ~200 nM), and in both conformations of the peptide, the sidechain of W7 is deeply buried into the hydrophobic cleft on the surface of S100B(ββ) and stabilizes the bound conformation of the peptide.

### 4.2. Energetics of S100B(ββ)–Peptide Complexes

**Figure 4:**
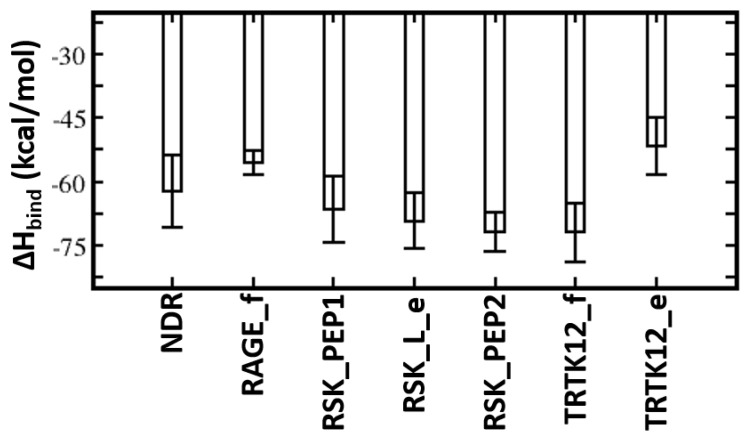
Energetic analysis of the molecular dynamics (MD) simulations of the S100B(ββ)–peptide complexes. Binding free energies of the interactions of S100B(ββ) with each peptide were calculated using the MMPBSA approach (see Methods) using the conformations sampled during the MD simulations of the S100B(ββ)–peptide complexes.

MMPBSA was used to quantify the binding affinities of these peptides with S100B(ββ). The TRTK12 peptide bound in an alpha helical conformation exhibited the highest binding affinity with ΔH of ~ −81 kcal/mol (Figure 4). Both the charge (R2, K4, K9) and hydrophobic (I5, W7, I10, L11) residues contribute favourably (>2 kcal/mol each) to the binding (Figure S4). Interestingly, the binding affinity of the same peptide was reduced by ~30 kcal/mol when it was bound in an extended coil conformation (TRTK12_e) (Figure 4). Given that this peptide is unstructured in the solution if it binds as an alpha helix upon binding, it will undergo conformational changes to adopt an alpha helical conformation, which will be associated with a large entropic penalty and is expected to negatively impact the binding of the peptide in the alpha helical conformation. In contrast, if the peptide binds in an extended coil conformation, the associated entropic penalty will be much lesser. Despite this, TRTK12 binds tighter, suggesting that the enthalpic interactions between peptide and S100B(ββ) dominate over entropic penalties incurred by the peptides upon binding. The RAGE peptide exhibited the least binding affinity (~−55 kcal/mol) amongst all the alpha helical peptides studied here. In the case of S100B(ββ)-RSK1, three different but overlapping fragments from RSK1 in complex with S100B(ββ) were simulated, and all three RSK1 fragments exhibited higher and similar binding affinities (~−70 kcal/mol). Interestingly, the RSK1 fragment, both in its alpha helical (RSK_PEP1 and RSK_PEP2) and extended conformations (RSK_L_e), has similar binding affinities. Unlike the TRTK12 and RAGE peptides, in which the binding is predominantly mediated by hydrophobic residues with added contributions from charged/polar residues, in the case of RSK1_PEP2 (helical region residues from 718 to 731), the contribution mostly arises from charged residues. Residues Q724, R725, R726, R728 and K729 along with residues I721, A723 and V727 from RSK1_PEP2 contribute favourably to the binding (Figure S4). In contrast, the binding is dominated by hydrophobic residues in the case of RSK1_PEP1 (helical region residues from 697 to 712). Hydrophobic residues A697, M698, A699, A704, L705, P710 and P712 and charged/polar residues T701, Y702, K709 and T711 from RSK_PEP1 contribute equally to the binding (Figure S4). The NDR peptide, which binds in a completely different orientation as compared to the other peptides, exhibited weaker binding affinity with ΔH of ~−62 kcal/mol (Figure 4). This peptide, despite being alpha helical, is not amphipathic and has hydrophobic residues exposed to solvent and charged/polar residues occupying the hydrophobic pocket. Therefore, only a subset of charged/polar residues (K72, R78, K80, R81 and R83) and hydrophobic residues (F76, L77, L79, L84) contribute favourably, while the other residues (E73, E75, E87) do not engage in favourable contributions to the binding (Figure S4).

### 4.3. In Silico Optimization of S100B(ββ) Binding Peptides/Design of Consensus Peptide

All the peptides studied above share an overlapping interaction surface on S100B(ββ), but share little sequence similarity, adopt different secondary structures and bind with diverse affinities (from nanomolar to micromolar) to S100B(ββ). One way to improve the binding is to derive a consensus sequence that retains the residues that contribute favourably and replace the residues that make unfavourable contributions with residues that are known to contribute favourably.

**Figure 5:**
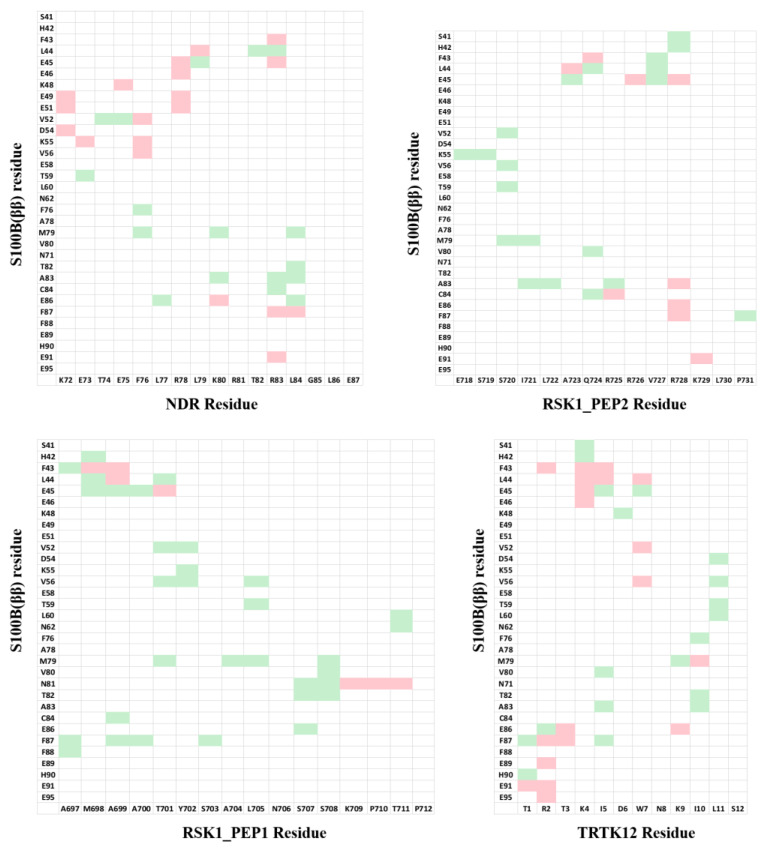
Pairwise residue decomposition of the binding energies of the S100B(ββ)-peptide complexes. The pairwise residue contributions were calculated with the MMPBSA approach (see Methods) using the conformations sampled during the MD simulations. Residue pairs that contribute favourably (green: 1 to 2.5 kcal/mol; red > 2.5 kcal/mol) to the interactions are highlighted.

For our strategy, instead of looking at the individual residues from the peptides, we looked at the pairwise residue interactions from S100B(ββ) and peptides that contribute favourably to the binding. The pairwise residue contribution analysis helped us to understand the nature of the interactions between the residues from S100B(ββ) and the peptide. This information can be used to incorporate appropriate amino acids on the peptide to gain additional interactions with the S100B(ββ) protein. The pairwise residue contribution was calculated using MMPBSA for the S100B(ββ)-NDR, S100B(ββ)-RSK_PEP1, S100B(ββ)-RSK_PEP2, S100B(ββ)-TRTK12, S100B(ββ)-RAGE and S100B(ββ)-p53 complexes (data taken from our previous work 61) (Figure 5). Data generated from these six S100B(ββ)-peptide complexes were used to optimize the sequences of four S100B(ββ)-peptide (NDP/RSK_PEP1/RSK_PEP2/TRTK12) complexes.

**Figure 6:**
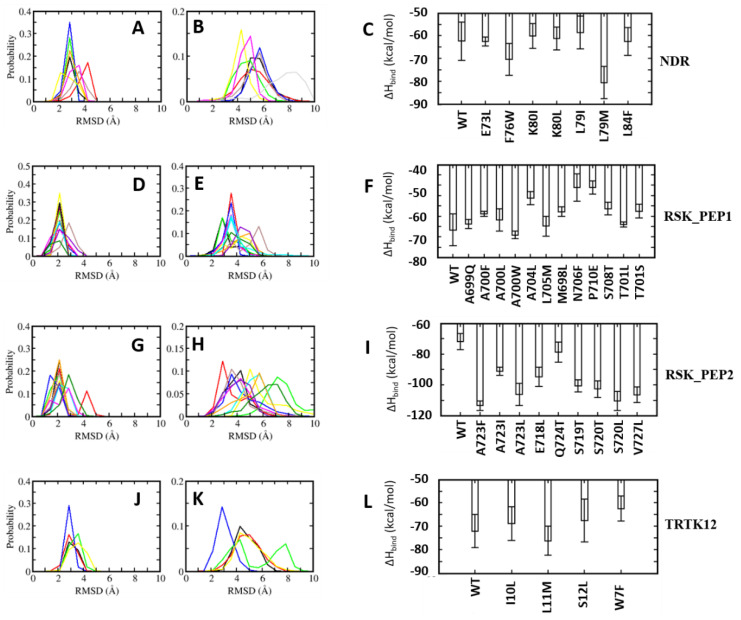
Distribution of root mean square deviation (RMSD) of (**A**,**D**,**G**,**J**) S100B(ββ) and (**B**,**E**,**H**,**K**) the bound peptides ((**A**,**B**): S100B(ββ)-NDR; (**D**,**E**): S100B(ββ)-RSK_PEP1; (**G**,**H**): RSK_PEP2; (**J**,**K**): S100B(ββ)-TRTK12 complexes). Different colours correspond to the different mutations in the peptide (NDR (black: WT, red: E73L, green: F76W, blue: K80I, yellow: K80L, brown: L79I, magenta: L79M, grey: L84F), RSK_PEP1 ( black: WT, red: A699Q, green: A700F, blue: A700L, yellow: A700W, brown: A704L, magenta: L705M, orange: M698L, cyan: N706F, violet: P710E, dark green: S708T, turquoise: T701L, maroon: T701S), RSK_PEP2 (black: WT, red: A723F, green: A723I, blue: A723L, yellow: A723W, brown: E718L, cyan: Q724T, magenta: S719T, orange: S720I, violet: S720L, dark green: V727L), TRTK12 (black: WT, red: I10L, green: L11M. blue: S12L, orange: W7F)). (**C**,**F**,**I**,**L**) Binding free energies of the interactions of S100B(ββ) with each peptide were calculated with the MMPBSA approach (see Methods) using the conformations sampled during the MD simulations of the S100B(ββ)-stapled peptide complexes.

In the case of the NDR peptide, several point mutations, E73L, F76W, L79M, K80L and L84F, were introduced in the linear peptide. The mutations were introduced upon detailed analyses of the contributions of the resides to the binding energies. The residue E73 NDR did not contribute favourably to the binding of the peptide (Figure 5); pairwise decomposition analysis of the other S100B(ββ)-peptide complexes suggests that S100B(ββ) residues (V52, V56, K55, T59) in the vicinity of the NDR E73 engage in favourable packing interactions with hydrophobic residues in the bound peptides (I721 from RSK_PEP1; L705, T701, Y702 from RSK1_PEP2; W7, L11 from TRTK12; L64, W61 from RAGE; M384, H380 from p53). Therefore, E73 was substituted with L in the NDR^E73L^ peptide. Similarly, F76 was substituted with W, L79 was substituted with M and I, K80 was substituted with I and L and L84 was substituted with F, resulting in the mutant peptides NDR^F76W^, NDR^L79M^, NDR^L79I^, NDR^K80I^, NDR^K80L^ and NDR^L84F^. MD simulations were carried out on the complexes of these mutant peptides complexed to S100B(ββ). During the simulations, the complexes remained stable, with no unbinding of any of the peptides observed. The S100B(ββ) dimer also remained stable, with RMSD < 4 Å from the experimental structure (Figure 6A). The bound mutant peptides also remained stable, but with increased flexibility, as was also seen for the complex with the NDR^WT^ peptide. NDR^E73L^, NDR^K80I^ and NDR^L79I^ had RMSD patterns similar to NDR^WT^, whereas NDR^F76W^, NDR^K80L^ and NDR^L79M^ exhibited greater stability (Figure 6B). The NDR^L84F^ peptide was the most flexible, with RMSD ~8 Å (Figure 6B). Binding energies of the mutant peptides using MMPBSA suggested binding similar to NDR^WT^ (Figure 6C). However, two of the three peptides that exhibited increased stability as compared to NDR^WT^, NDR^F76W^ and NDR^L79M^ displayed improved binding affinities by ~8 and ~18 kcal/mol, respectively (Figure 6C).

A similar exercise was carried out for the other three peptides. In the RSK1_PEP1^WT^ peptide, residues Met698, Ala699, Thr701 and Leu705 contribute favourably and residues A700 and S708 make negligible contributions to binding (Figure S4). Examination of the residue pairwise analysis of the other S100B(ββ)-peptide complexes suggested that these residues can be replaced by large and bulky hydrophobic residues or polar residues for improving binding affinities (Figure 5). This guided us to construct the following mutations: Met698 was replaced by Leu; Ala699 was replaced by Gln; Ala700 was replaced by hydrophobic residues Leu, Phe and Trp; Thr701 was replaced by Leu and Ser; Ala704 was replaced by Leu; Leu705 was replaced by Met; Asn706 was replaced by Phe; Ser708 was replaced Thr; and Pro710 was replaced by Glu. MD simulations were carried out on the complexes of all these mutant peptides complexed to S100B(ββ). All the S100B(ββ)-RSK1_PEP1^MUTANT^ peptide complexes remained stable with no unbinding of peptides observed (Figure 6D,E). Similar to the wildtype, all the bound mutant peptides also remained stable with RMSD ~4 Å from the starting conformation (Figure 6E). Peptides RSK1_PEP1^L705M^ and RSK1_PEP1^S708T^ exhibited reduced stability with RMSD of 6 and 4.5 Å, respectively, compared to RSK1_PEP1 ^WT^, which had an RMSD of ~3.5 Å (Figure 6E). The corresponding S100B(ββ) dimer also remained stable within RMSD < 4 Å from the experimental structure (Figure 6D). Although most of the mutations were structurally well tolerated, not all resulted in improved affinities (Figure 6F). Only peptide RSK1_PEP1^A700W^ displayed improved affinity as compared to the wildtype peptide. The other peptides were either similar to the wildtype (RSK1_PEP1^A699Q^, RSK1_PEP1^A700L^ and RSK1_PEP1^L705M^) or exhibited reduced affinities (RSK1_PEP1^A700F^, RSK1_PEP1^A704L^, RSK1_PEP1^M698L^, RSK1_PEP1^N7069F^, RSK1_PEP1^P710E^, RSK1_PEP1^S708T^ and RSK1_PEP1^T701S^) with ΔΔH ranging from 8 to 20 kcal/mol (Figure 6F).

In the case of RSK1_PEP2, residues that either contribute unfavourably (E718) or make negligible contributions (S719 and S720), and make favourable contributions (A723, Q724 and V727) (Figure S4), were identified to generate mutations that could result in improved binding affinities. Based on the analyses of the pairwise residue contributions, Glu718 was replaced by Leu; Ser719 was replaced by Thr; Ser720 was replaced by hydrophobic residues Ile and Val; Ala723 was replaced by hydrophobic residues Ile, Leu and Phe; Gln724 was replaced by Thr; and Val727 was replaced by Leu. MD simulations of the complexes of the mutant peptides with S100B(ββ) were stable (Figure 6G,H), with no unbinding of the peptides observed, and most of the bound mutant peptides remaining stable with RMSD ~4Å from the starting conformation (Figure 6H). RSK1_PEP2^A723F^, RSK1_PEP2^A723L^ and RSK1_PEP2^E718L^ displayed increased stability as compared to the wildtype peptide (Figure 6H); RSK1_PEP2^S719T^ and RSK1_PEP2^S720L^ displayed stability similar to the wildtype peptide; RSK1_PEP2^A723I^, RSK1_PEP2^S720I^, RSK1_PEP2^Q724T^ and RSK1_PEP2^V727L^ displayed decreased stability (RMSD ~5 to 8 Å) as compared to the wildtype peptide (RSMD ~4 Å). (Figure 6H) Interestingly, all the RSK1_PEP2^MUTANT^ peptides exhibited increased binding affinity as compared to the wildtype peptide, with RSK1_PEP2^Q724T^ exhibiting the smallest increase (~8 kcal/mol), while the rest resulted in increases of ~20–30 kcal/mol relative to the wildtype peptide, RSK1_PEP2^A723F^ exhibiting the highest gain (~40 kcal/mol) (Figure 6I). The increase in binding affinity correlated well with the hydrophobicity of the mutation (Figure 6I).

In the case of the TRTK12 peptide, the tightest binding peptide of S100B(ββ), most residues contribute favourably to binding (Figure S4). Thr3 from the disordered N-terminus and Asn8 and Ser12 from the alpha helical region of the peptide make negligible contributions, and analysis of the pairwise decomposition of the energetics suggested that Ser12 and some other residues can be replaced by residues from the other S100B(ββ)-peptide complexes, resulting in higher affinities (Figure 5). Ile10 and Leu11, which already contribute favourably to binding, were replaced by Leu and Met, respectively; Ser12 was replaced by Leu; Trp7, which anchors the peptide on to the surface of S100B(ββ), was replaced by Phe. MD simulations of the complexes of the mutant peptides with S100B(ββ) were stable (Figure 6J,K), with no unbinding of peptides observed. Similar to the wildtype TRTK12 peptide, most of the bound mutant peptides also remained stable with RMSD ~5 Å from the starting conformation (Figure 6K). TRTK12^S12L^ peptide displayed improved stability (RMSD ~3 Å) as compared to the wildtype TRK12 peptide (RMSD ~4 Å), whereas TRTK12^L11M^ displayed two peaks at ~4 and 8 Å, with the first peak corresponding to the peak observed for the wildtype peptide (Figure 6K). Interestingly, TRTK12^W7F^ also displayed stability similar to the wildtype peptide. The corresponding S100B(ββ) conformations also remained stable with RMSD < 4 Å (Figure 6J). Unsurprisingly, TRTK12^W7F^ displayed reduced binding (by ~10 kcal/mol) as compared to the wildtype peptide (Figure 6L), highlighting the importance of the indole sidechain, while TRTK12^L11M^ exhibited improved binding (by ~4 kcal/mol) as compared to the wildtype peptide (Figure 6L); the other mutant peptides TRTK12^I10L^ and TRTK12^S12L^ displayed binding affinities similar to the wildtype peptide (Figure 6L).

### 4.4. Design of Stapled Peptide Binders of S100B(ββ)

**Figure 7:**
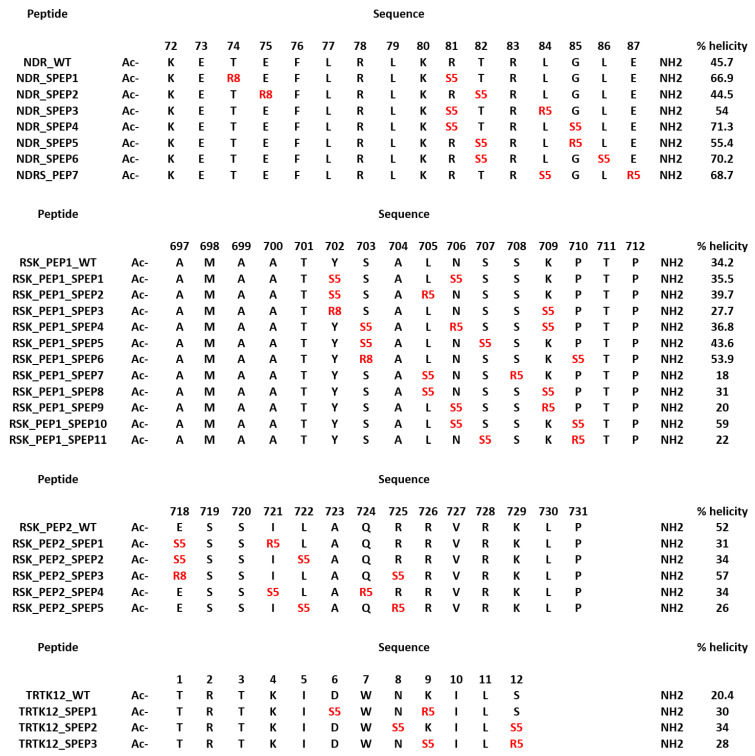
Sequences of the designed stapled peptides. Residues that are linked through all hydrocarbon linkers i,i+3, i,i+4 and i,i+7 are highlighted in red. The helicity (percentage) of peptides when bound to S100B(ββ) are shown.

In parallel to the design of the mutant peptides, we also designed stapled peptides based on the S100B(ββ) binding linear peptides. Despite being predominantly disordered, several S100B(ββ) binding peptides (TRTK12, RSK_PEP1, RSK_PEP2, NDR and p53) adopt alpha helical conformations upon binding to S100B(ββ). Constraining the peptide in its bound state alpha helical conformation could yield better binding S100B(ββ) peptides. We used stapling to constrain the peptides; in peptide stapling, RCM or click chemistry is used to link the side chains of two unnatural amino acids that are strategically positioned, where the linkers can be of different lengths and/or with functional groups attached. The unnatural amino acids are usually introduced at positions where the peptide residues are not critical for binding. We identified these positions by analysing the contributions of each residue to the total binding energy (Figure S4). In the case of the NDR peptide, residues Thr74, Glu75, Thr82, Gly85, Leu86 and Glu87 make negligible or unfavourable contributions; Arg81 and Leu84 make favourable contributions yet are exposed to the solvent. In the case of RSK_PEP1, residues at positions Tyr702, Ser703, Leu705, Asn706, Ser707, Ser708, Lys709, Pro710 and in RSK_PEP2 residues at positions Glu718, Ile721, Leu722, Gln724 and Arg725 were identified as either contributing negligibly or unfavourably to the overall binding energies or contributing favourably and yet pointing into the solvent. In the case of the TRTK12 peptide, residues Asp6, Asn8, Lys9 and Ser12 from the alpha helix were identified as positions where the staple linkers could be introduced. Based on the spatial distances between the pairs of residues, i,i+3 or i,i+4 or i,i+7 staple linkers were introduced. The details of the designed stapled peptides are shown in Figure 7. The model of S100B(ββ)-stapled peptide complexes were generated by incorporation of the hydrocarbon linkers in the peptides in the presence of bound S100B(ββ).

**Figure 8:**
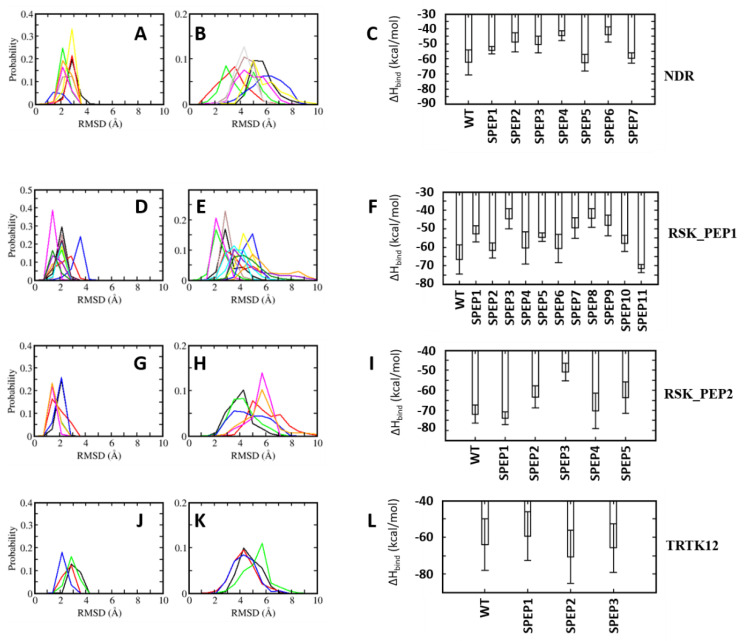
Distribution of root mean squared deviations (RMSD) of (**A**,**D**,**G**,**J**) S100B(ββ) and (**B**,**E**,**H**,**K**) the bound stapled peptides ((**A**,**B**): S100B(ββ)-NDR; (**D**,**E**): S100B(ββ)-RSK_PEP1; (**G**,**H**): RSK_PEP2; (**J**,**K**): S100B(ββ)-TRTK12 complexes). Different colours correspond to different stapled peptides ((NDR: black: WT, red: SPEP1, green: SPEP2, blue: SPEP3, yellow: SPEP4, brown: SPEP5, magenta: SPEP6, grey: SPEP7) (RSK_PEP1: black: WT, red: SPEP1, green: SPEP2F, blue: SPEP3, yellow: SPEP4, brown: SPEP5, magenta: SPEP6, orange: SPEP7, cyan: SPEP8, violet: SPEP9, dark green: SPEP10, turquoise:SPEP11) (RSK_PEP2: black: WT, red: SPEP1, green: SPEP2F, blue: SPEP3, orange: SPEP4, magenta: SPEP5) (TRTK12: black: WT, red: SPEP1, green: SPEP2F, blue: SPEP3)). (**C**,**F**,**I**,**L**) Binding free energies of the interactions of S100B(ββ) with each stapled peptide were calculated with the MMPBSA approach (see Methods) using the conformations sampled during the MD simulations of the S100B(ββ)-stapled peptide complexes.

All the complexes of S100B(ββ) with the stapled peptides were subject to MD simulations. During the MD simulations, all the modelled S100B(ββ)-NDR stapled peptide (NDR_SPEP) complexes remained stable with no unbinding observed for any of the S100B(ββ)-NDR_SPEP complexes (Figure 8A,B). The peptide bound conformations of the S100B(ββ) dimer were stable with RMSD ~3 Å during the simulations (Figure 8A). Most of the bound stapled peptides displayed improved stability with RMSD ~< 4 Å as compared to their unstapled linear counterparts (RMSD ~5–6 Å), except NDR_SPEP3, which displayed increased flexibility upon stapling (Figure 8B). Secondary structural analyses showed that in comparison to the unstapled linear peptides which exhibited 58% helicity in the bound states, the stapled peptides displayed varying levels of helicity ranging from 44% to 71% in their bound states (Figure 7). Binding energies of the S100B(ββ)-stapled peptide complexes showed that in comparison to the unstapled linear peptides, most of the stapled peptides displayed reduced binding energies by ~6 to 20 kcal/mol (Figure 8C). This suggests that increasing the conformational rigidity of the peptides by stapling results in loss of flexibility that is clearly required for stable binding. Only NDR_SPEP5 and NDR_SPEP7 exhibited binding energies similar to the wildtype, suggesting tolerance for the staple linker in these two peptides (Figure 8C).

In the case of RSK_PEP1-stapled peptide (RSK_PEP1_SPEP) complexes, most of the stapled peptides exhibited increased flexibility upon stapling, except for RSK_PEP1_SPEP2 and RSK_PEP1_SPEP5, which displayed improved stability upon stapling, and RSK_PEP1_SPEP6, which displayed similar stability as the wildtype peptide (Figure 8D,E). In contrast, the peptide bound S100B(ββ) remained stable with RMSD <~3 Å (Figure 8D). Both the N- and C-termini of the unstapled linear RSK_PEP1 peptide remained disordered/unstructured even in its bound state; therefore, the unstapled RSK_PEP1 peptide exhibited reduced helicity (~35%) even in the bound state (Figure 7). The stapled peptides exhibited varying levels of helicity ranging from 18% to 60%, suggesting that the stapling had constrained the alpha helical conformations of this peptide. Binding energy calculations suggested that the affinities of RSK_PEP1_SPEP1, RSK_PEP1_SPEP5, RSK_PEP1_SPEP7, RSK_PEP1_SPEP8 and RSK_PEP1_SPEP9 were reduced by ~20 kcal/mol as compared to the unstapled linear counterpart (Figure 8F), while RSK_PEP1_SPEP2, RSK_PEP1_SPEP4, RSK_PEP1_SPEP6 and RSK_PEP1_SPEP10 retained the binding affinity of wildtype (Figure 8F). Only RSK_PEP1_SPEP11 displayed a small increase in affinity (~5 kcal/mol) as compared to the unstapled RSK_PEP1 peptide; this peptide exhibited reduced helicity (18% compared to 34% in the wildtype) (Figure 7) when bound to S100B(ββ), once again highlighting the need for flexibility in the peptides for optimal interactions with S100B(ββ).

In the case of RSK_PEP2, a different region from RSK1 occupies the same pocket that was occupied by the other S100B(ββ) binding peptides. The bound linear peptide exhibited 52% helicity (Figure 7). Based on the per-residue decomposition analysis, five stapled peptides were designed and simulated in complex with S100B(ββ). Three of the stapled peptides (RSK_PEP2_SPEP1, RSK_PEP2_SPEP4 and RSK_PEP2_SPEP5) displayed decreased stability (RMSD ~ 5–6 Å), and the other two stapled peptides (RSK_PEP2_SPEP3 and RSK_PEP2_SPEP4) displayed similar stability (RMSD ~4 Å) compared the wildtype peptide (Figure 8H). The bound stapled peptides also displayed varying levels of helicity ranging from 26% to 57% in their bound states (Figure 7). The corresponding peptide bound S100B(ββ) remained stable, similar to the wildtype peptide bound S100B(ββ) with RMSD ~2–3 Å (Figure 8G). Four of the five stapled peptides had binding affinities similar to the unstapled linear peptide, while one peptide (RSK_PEP2_SPEP3), which displayed similar stability as compared to the wildtype peptide, exhibited reduced binding by ~20 kcal/mol (Figure 8I). This peptide, RSK_PEP2_SPEP3, exhibited increased helicity in its bound states upon stapling, and the associated reduction in affinity again highlights the need for flexibility in the peptides for improved affinities.

TRTK12 is a small alpha helical peptide and a tight binder of S100B(ββ). Based on the positions that were identified using the per-residue decomposition analysis, three stapled peptides (TRTK12_SPEP) were designed and simulated in complex with S100B(ββ). All the stapled peptides displayed similar stability profiles (RMSD ~4 Å) (Figure 8K), and were similar to the wildtype peptide; the peptide bound S100B(ββ) also remained stable during the MD simulations (Figure 8J). The unstapled linear peptide exhibited low helicity (~20%) when bound to S100B(ββ), and all the stapled TRTK12 peptides exhibited increased helicity by 10% (Figure 7), but retained affinities similar to that of the wildtype, with only TRTK12_SPEP2 exhibiting increased affinity (by 5 kcal/mol; Figure 8L).

### 4.5. Optimization of Stapled Peptides through In Silico Mutagenesis

So far, we have identified positions in the linear peptides that could be replaced by other amino acids, guided by the complexes of the other S100B(ββ) binding peptides that occupy the same binding pocket on the surface of S100B(ββ). Some of the designed peptides resulted in improved binding energies to S100B(ββ) as compared to the corresponding wildtype peptides. We also designed several stapled peptides that had either improved affinity or retained the binding affinity to S100B(ββ) as compared to the corresponding wildtype peptides. Next, we combined both the mutations and peptide stapling to investigate whether the combinations resulted in improved affinities as compared to the unstapled linear counterparts. We chose to combine only the mutations and the stapled peptides that were shown (above) to either retain or improve the binding affinities to S100B(ββ). Details of the mutated stapled peptides are given in Figure S5. The models of the S100B(ββ)–SPEP_MUTANTs were generated based on the structures of the corresponding S100B(ββ)–unstapled peptide complexes and were subjected to MD simulations.

**Figure 9:**
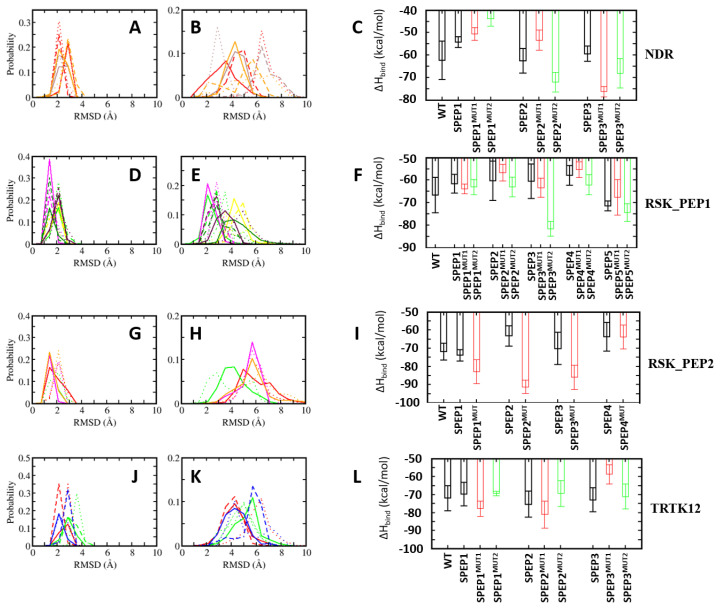
Distribution of root mean squared deviations (RMSD) of (**A**,**D**,**G**,**J**) S100B(ββ)and (**B**,**E**,**H**,**K**) the bound stapled mutant peptides ((**A**,**B**): S100B(ββ)–NDR; (**D**,**E**): S100B(ββ)–RSK_PEP1; (**G**,**H**): RSK_PEP2; (**J**,**K**): S100B(ββ)-TRTK12 complexes). Different colours correspond to the different stapled wildtype peptides (continuous) and stapled mutant peptides ((MUT/MUT1: dashed; MUT2: dotted lines) (NDR: red: SPEP1, orange: SPEP2, grey: SPEP3) (RSK_PEP1: green: SPEP1, yellow: SPEP2, magenta: SPEP3, dark green: SPEP4, maroon: SPEP5) (RSK_PEP2: red: SPEP1, green: SPEP2, orange: SPEP3, magenta: SPEP4) (TRTK12: red: SPEP1, green: SPEP2, blue: SPEP3)). (**C**,**F**,**I**,**L**) Binding free energies of the interactions of S100B(ββ) with each stapled mutant peptide were calculated with the MMPBSA approach (see Methods) using the conformations sampled during the MD simulations of the S100B(ββ)–stapled mutant peptide complexes.

In the case of NDR_SPEP peptides, two different sets of peptides were designed. In the first set (MUT2), two mutations (F76W and L79M) were combined with the stapled peptides (NDR_SPEP1, NDR_SPEP5 and NDR_SPEP7). In the second set (MUT5), in addition to these two mutations, three additional mutations (E73L, K80L and L84F) were incorporated in each of the three stapled peptides (NDR_SPEP1, NDR_SPEP5 and NDR_SPEP7). In the case of NDR_SPEP1, both the double mutant (NDR_SPEP1^MUT2^) and penta mutant (NDR_SPEP1^MUT5^) peptide resulted in decreased stability with RMSD of ~5 and ~7 Å as compared to RMSD of ~3 Å for the wildtype stapled peptide (Figure 9B). The NDR_SPEP1^MUT2^ peptide exhibited reduced helicity of ~59%, and NDR_SPEP1^MUT5^ retained helicity (~68%) as compared to wildtype peptide (~67% helicity) in its bound state (Figure S5). In contrast the peptide, bound S100B(ββ) did not show any major differences across the simulations (Figure 9A). Both mutant NDR_SPEP1 peptides resulted in decreased binding by 5–10 kcal/mol (Figure 9C). In the case of NDR_SPEP5, the double mutant peptide NDT_SPEP5^MUT2^ showed increased flexibility, and the five mutant peptide NDR_SPEP5^MUT5^ displayed reduced flexibility as compared to the wildtype stapled peptide NDR_SPEP2 (Figure 9B). Both the mutant peptides NDT_SPEP5^MUT2^ and NDT_SPEP5^MUT5^ retained helicity at ~57% and 49%, similar to the wildtype NDR_SPEP5 peptide (55%) (FigureS49). The NDR_SPEP5^MUT2^ resulted in reduced binding by ~10 kcal/mol, whereas the NDR_SPEP2^MUT5^ displayed improved affinity by ~−10 kcal/mol (Figure 9C). In the case of NDR_SPEP7, both the mutant peptides, NDR_SPEP7^MUT2^ and NDR_SPEP7^MUT5^, exhibited slightly increased flexibility and also displayed reduced helicity (48% and 52% compared to 69% in wildtype) in their bound states (Figure S5). Both the peptides exhibited improved binding affinity to S100B(ββ) by ~15 kcal/mol and ~10 kcal/mol, respectively (Figure 9C), and the observation that they are less helical and more flexible again underscores the need for flexibility to improve affinity in this system.

In the case of RSK_PEP1_SPEP, two different sets of mutant peptide simulations were carried out. In the first set (MUT3), three mutations (A700W, T701L and L705M) and in the second set (MUT6) three additional mutations (M698L, A699Q, S708T) were combined with the stapled peptides (RSK_PEP1_SPEP2, RSK_PEP1_SPEP4, RSK_PEP1_SPEP6, RSK_PEP1_SPEP10 and RSK_PEP1_SPEP11). In the case of RSK_PEP1_SPEP2, both the mutant peptides, RSK_PEP1_SPEP2^MUT3^ and RSK_PEP1_SPEP2^MUT6^, displayed enhanced stability as compared to the corresponding wildtype stapled peptide (RSK_PEP1_SPEP2) (Figure 9E), and the helicity of the bound mutant peptides remained similar to the wildtype peptide (32% and 39%, respectively, for the RSK_PEP1_SPEP2^MUT3^ and RSK_PEP1_SPEP2^MUT6^ peptides compared to 36% for the RSK_PEP1_SPEP2 peptide) (Figure S5). Both the mutant peptides also exhibited slightly improved affinity (by ~3 kcal/mol) as compared to the RSK_PEP1_SPEP2 peptide (Figure 9F). In the case of RSK_PEP1_SPEP4, both RSK_PEP1_SPEP4^MUT3^ and RSK_PEP1_SPEP4^MUT6^ exhibited stability similar to the RSK_PEP1_SPEP4 peptide, but both these peptides resulted in much lesser helicity (~20%) as compared to the wildtype peptide (~ 37%) (Figure S5). Despite the reduced helicity, RSK_PEP1_SPEP4^MUT3^ resulted in ~8 kcal/mol less affinity, and RSK_PEP1_SPEP4^MUT6^ resulted in ~ 3 kcal/mol improved affinity (Figure 9F). Both mutant peptides of the RSK_PEP1_SPEP6 peptide (which exhibited ~54% helicity (Figure S5) in its bound state and bound stably to S100B(ββ) with RMSD ~ 2 Å) remained stably bound to S100B(ββ) with RMSD ~3 Å (Figure 9E) but exhibited reduced helicity (~ 42%) in its complex with S100B(ββ) (Figure S5). Both the mutant peptides displayed improved affinity to S100B, with RSK_PEP1_SPEP6^MUT3^ gaining ~4 kcal/mol and RSK_PEP1_SPEP6^MUT6^ gaining ~25 kcal/mol (Figure 9F). Peptide RSK_PEP1_SPEP10 displayed the highest helicity (60%) in its bound state amongst all the stapled RSK_PEP1 peptides, and both the mutant (RSK_PEP1_SPEP10^MUT3^ and RSK_PEP1_SPEP10^MUT6^) RSK_PEP1_SPEP10 peptides retained the helicity (~55%) in their bound states (Figure S5). Both the mutant peptides also displayed improved conformational stability (RMSD ~3 Å) compared to the wildtype (RMSD ~4 Å) stapled peptide RSK_PEP1_SPEP10 (Figure 9E). However, neither mutant peptide showed enhanced binding to S100B(ββ), with RSK_PEP1_SPEP10^MUT3^ peptide losing ~4 kcal/mol in affinity, while RSK_PEP1_SPEP10^MUT6^ peptide gained affinity but only by ~4 kcal/mol (Figure 9F). A similar pattern was also observed for the RSK_PEP1_SPEP11 peptide. This peptide was the least helical (22% helicity when bound to S100B(ββ)) and also displayed the highest binding affinity from the RSK_PEP1_SPEP series. RSK_PEP1_SPEP10^MUT3^ showed slightly increased flexibility (RMSD of ~5 Å), and RSK_PEP1_SPEP10^MUT6^ showed slightly increased stability (RMSD ~3 Å), as compared to the wildtype RSK_PEP1_SPEP10 peptide (RMSD ~4 Å) (Figure 9E). Both the mutant peptides also resulted in increased helicity (42% vs. 22% observed in the wildtype; Figure S5) when in complex with S100B(ββ). RSK_PEP1_SPEP10^MUT3^ lost some affinity for S100B (~4 kcal/mol), while RSK_PEP1_SPEP10^MUT6^ gained a little affinity (~4 kcal/mol) (Figure 9F).

In the case of RSK_PEP2_SPEP, five mutations (S719T, S720L, A723F, Q724T and V727L) that showed improved affinity were combined with the four stapled peptides (RSK_PEP2_SPEP1, RSK_PEP2_SPEP2, RSK_PEP2_SPEP4 and RSK_PEP2_SPEP5) that retained binding to S100B(ββ), similar to the wildtype stapled peptides. All four mutant stapled peptides (RSK_PEP2_SPEP1^MUT^, RSK_PEP2_SPEP2^MUT^, RSK_PEP2_SPEP4^MUT^ and RSK_PEP2_SPEP5^MUT^) displayed stability profiles that were very similar to the corresponding wildtype stapled peptides in the bound states (Figure 9G,H); the corresponding bound S100B also exhibited very similar stability profiles (Figure 9G). However, the helicity of the mutants varied slightly as compared to their wildtype stapled counterparts. Peptide RSK_PEP2_SPEP1^MUT^ retained helicity (35%) similar to the wildtype stapled peptide (31%), and peptides RSK_PEP2_SPEP2^MUT^ and RSK_PEP2_SPEP5^MUT^ showed increased helicity (~49% and ~33% as compared to its wildtype staple peptides at ~34% and ~ 26%, respectively) (Figure S5). RSK_PEP2_SPEP4^MUT^ had reduced helicity of ~19% compared to ~34% of the wildtype stapled peptide (Figure S5). Despite the differences in helicity between the mutant and wildtype stapled peptides, three mutant peptides, RSK_PEP2_SPEP1^MUT^, RSK_PEP2_SPEP2^MUT^ and RSK_PEP2_SPEP4^MUT^, displayed enhanced binding by ~10, ~25 and ~15 kcal/mol, respectively (Figure 9I). The RSK_PEP2_SPEP5^MUT^ peptide retained affinity similar to its wildtype stapled peptide counterpart (Figure 9I).

In the case of TRTK12, only one mutation (L11M) was shown to improve it binding affinity, and two other mutations (I10L, S12L) were shown to retain the affinity for S100B(ββ). All three stapled peptides were shown to retain binding to S100B(ββ). Therefore, two sets of mutant simulations were carried out: in the first set (MUT1), only one mutation (L194M) was combined with the stapled peptides TRTK12_SPEP. In the second set (MUT3), all three mutations were combined together with the stapled peptides TRTK12_SPEP. Only in the case of TRTK12_SPEP1, all three mutations were introduced, while in the other two cases (TRTK12_SPEP2 and TRTK12_SPEP3), only two mutations (L11M and I10L) (MUT2) were combined, as the position of the third mutation (S12) was used for the incorporation of the staple linker. In the case of TRTK12_SPEP1, the triple mutant peptide TRTK12_SPEP1^MUT3^ displayed a stability profile similar to the WT staple peptide TRTK12_SPEP1 (RMSD ~4 Å) (Figure 9K), whereas the single mutant peptide TRTK12_SPEP1^MUT1^ exhibited increased flexibility with RSMD ~6 Å (Figure 9K). Both the mutant peptides also exhibited different helicities (~29% and 20%, respectively, for TRTK12_SPEP1^MUT1^ and TRTK12_SPEP1^MUT3^), compared to the wildtype stapled peptide (~30% helicity) when bound to S100B(ββ) (Figure S5). The peptide TRTK12_SPEP1^MUT1^ with single mutation displayed improved affinity (~5 kcal/mol), whereas the TRTK12_SPEP1^MUT3^ peptide retained affinity similar to the wildtype stapled peptide TRTK12_SPEP1 (Figure 9L). In the case of TRTK12_SPEP2 peptide, both the single and double mutant peptides, TRTK12_SPEP2^MUT1^ and TRTK12_SPEP2^MUT2^, exhibited enhanced stabilities (Figure 9K) as compared to the wildtype TRTK12_SPEP2 peptide and also retained helicity (~33% and 31%, respectively, for the TRTK12_SPEP2^MUT1^ and TRTK12_SPEP2^MUT3^, similar to the wildtype stapled peptide TRTK12_SPEP2 at ~34%) (Figure S5). The TRTK12_SPEP2^MUT1^ peptide displayed slightly improved affinity (~5 kcal/mol), whereas the TRTK12_SPEP2^MUT2^ peptide displayed reduced affinity (~5 kcal/mol) as compared to the wildtype peptide TRTK12_SPEP2 (Figure 9L). In the case of the TRTK12_SPEP3 peptide, the single mutant peptide TRTK12_SPEP2^MUT1^ displayed a stability profile similar to the wildtype TRTK12_SPEP3 peptide with RMSD ~4 Å, whereas the TRTK12_SPEP3^MUT2^ peptide displayed decreased stability with RMSD ~6 Å (Figure 9K). However, the single mutant peptide TRTK12_SPEP3^MUT1^ resulted in reduced helicity (~17%), and the double mutant peptide TRTK12_SPEP3^MUT2^ retained helicity of ~28%, similar to the wildtype peptide TRTK12_SPEP3 (Figure S5). In contrast, the single mutant peptide resulted in reduced binding (by ~15 kcal/mol), whereas the double mutant peptide retained affinity similar to the wildtype stapled peptide TRTK12_SPEP3 (Figure S5).

### 4.6. Design of Stapled Peptide-Based Imaging Probes for Detection of Biomarker S100B(ββ)

**Figure 10:**
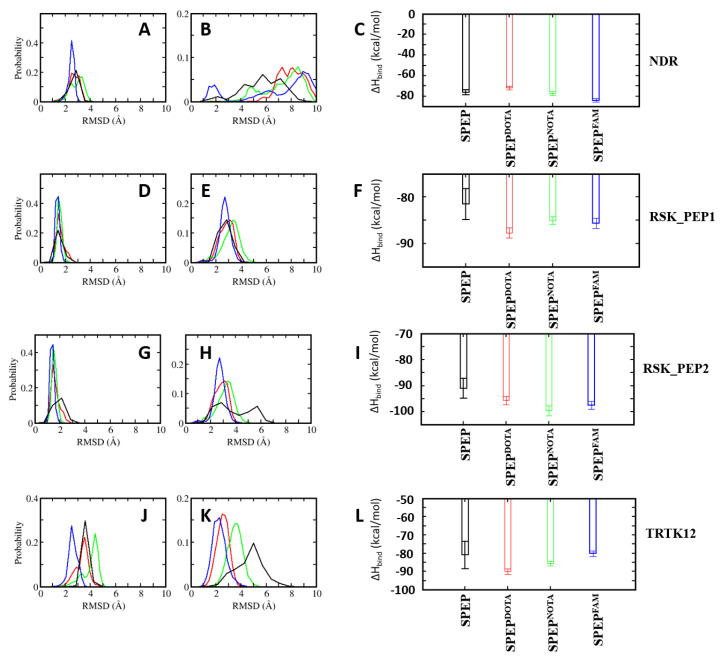
Distribution of root mean squared deviations (RMSD) of (**A**,**D**,**G**,**J**) S100B(ββ) and (**B**,**E**,**H**,**K**) the bound stapled peptides conjugated with imaging probes (DOTA, NOTA and FAM) ((**A**,**B**): S100B(ββ)–NDR; (**D**,**E**): S100B(ββ)–RSK_PEP1; (**G**,**H**): RSK_PEP2; (**J**,**K**): S100B(ββ)-TRTK12 complexes). Different colours correspond to the stapled peptides with different probes (black: no probe, red: DOTA, green: NOTA and blue: FAM). (**C**,**F**,**I**,**L**) Binding free energies of the interactions of S100B(ββ) with the stapled peptide probes were calculated with the MMPBSA approach (see Methods) using the conformations sampled during the MD simulations of the S100B(ββ)–stapled peptide probe complexes.

In addition to being associated with cancer, the upregulated levels of S100B(ββ) are used as a prognostic indicator for assessing disease progression, disease recurrence and metastatic potential in patients with malignant melanoma, traumatic brain injury (TBI) and brain metastasis, and hence can be a good biomarker for early detection and treatment. In an effort to design molecular probes for the detection of the S100B(ββ) protein, we used the S100B(ββ) stapled peptides conjugated with the imaging agents DOTA, NOTA and FAM. From the structural analysis of S100B(ββ)–stapled peptide complexes, we identified that the N-terminal of the peptide can be used to incorporate imaging agents. We used the four best mutant stapled peptides identified in this study, one each from NDR_SPEP7^MUT2^, RSK_PEP1_SPEP6^MUT6^, RSK_PEP2_SPEP2^MUT^ and TRTK12_SPEP2^MUT1^, and added probes at their N-termini; for each peptide, we modelled the respective S100B(ββ)–SPEP:DOTA, S100B(ββ)–SPEP:NOTA and S100B(ββ)-SPEP:FAM complexes. To avoid any potential clashes between the probes with the surface of S100B(ββ), the probes (DOTA, NOTA and FAM) were linked to the N-termini of the peptides with a PEG200 linker. All the modelled complexes were subjected to MD simulations. During the simulations, all the stapled peptides conjugated with the imaging agents remained bound, and the stability profiles (RMSD) of the bound stapled peptides were very similar to the corresponding parent peptides (without the imaging agents) (Figure 10). In the case of S100B(ββ)-RSK1_PEP2_SPEP2^MUT^ and S100B(ββ)-TRTK12_SPEP2^MUT1^, the imaging agent conjugated peptides displayed enhanced stability as compared to their parent peptides (Figure 10). Similarly, all the imaging agent conjugated peptides retained binding affinities very similar to their corresponding optimized parent stapled peptides (Figure 10C). Our combined structural and energetic analysis suggests that the incorporation of probes at the N-termini of stapled peptides will not interfere with the binding of peptides to S100B(ββ) and, therefore, can be used as peptide-based probes for diagnosis.

## 5. Discussion

S100B(ββ) protein belongs to a family of multifunctional proteins that regulate a wide variety of cellular processes through their interactions with effector proteins. Overexpression of S100B(ββ) protein is associated with several human diseases, such as cancer, inflammatory disorders and neurodegenerative conditions. S100B(ββ) is also a clinically validated marker for malignant melanoma (elevated only in tumours), traumatic brain injury (TBI) [19,20,21,22,23] and has been associated with cancer progression in multiple myeloma, NSCLC/SCLC and pancreatic cancer [24,25,26,27,28]. Despite being validated as a therapeutic target, S100B(ββ) remains undruggable with no approved drugs in clinics and no candidate molecules in pre-clinical development. Continued efforts with the development of small molecule inhibitors for the disruption of the interactions of S100B(ββ) with partner proteins has resulted in limited success due to challenges associated with targeting PPIs with small molecules. Therefore, therapies targeting S100B(ββ) remain an unmet medical need.

Peptides and peptidomimetics have been shown to be very good inhibitors of PPIs and are increasingly of great interest because of recent successes in ensuring enhanced conformational and proteolytic stability and cell permeability using modifications such as stapling. We designed a set of stapled peptides using available experimental structures of S100B(ββ) bound with peptides from partner proteins. The stapled peptides derived from different protein partners displayed varying binding energies, with several displaying improved binding as compared to their respective linear counterparts. Our in silico mutagenesis study (mutations identified using the residue pairwise energy decomposition analysis) also identified several mutations that resulted in improved binding. Further optimization by combining the stapled peptides (identified using in silico staple scans) that exhibited improved binding with the mutations (identified using in silico mutagenesis studies) resulted in stapled peptides with improved binding affinities. The data suggest that the tolerability of mutations depends on the flexibility of the peptide molecules. We further designed stapled peptides conjugated to imaging robes that could be used for the detection of S100B(ββ) by taking the best binding stapled peptides and incorporating three widely used imaging agents, DOTA, NOTA and FAM. The affinities of these conjugated stapled peptide probes for S100B(ββ) were similar to those of the respective stapled peptides.

In summary, we designed a set of stapled peptides (probes), with improved affinities for S100B(ββ). These peptides have the potential to disrupt the biologically functional complexes of S100B(ββ) and could be used as lead molecules for the development of novel therapeutics against aberrant complexation. Simultaneously, these peptide-based probes can be used as diagnostic tools for the detection of the S100B(ββ) protein. In conclusion, the stapled peptide-based inhibitors and probes designed here could serve as good starting points for the further development of S100B(ββ) theranostics.

## Data Availability

The data presented in this study are available in the Supplementary Materials.

## Supplementary Materials

The following are available online.

## Sample Availability

Not available.
