# Supplementary material for: Computational Design of Macrocyclic Binders of S100B(ββ): Novel Peptide Theranostics
Source: Molecules. 2021 Jan 30;26(3):721. doi: 10.3390/molecules26030721 (PMC7866529; doi:10.3390/molecules26030721)
Supplement: Supplementary file 1 [file molecules-26-00721-s001.pdf]

Supplementary Information

Computational Design of Macrocyclic Binders of S100B(ββ): Novel Peptide Theranostics

Srinivasaraghavan Kannan [1,*], Pietro G. A. Aronica [1], Thanh Binh Nguyen [1], Jianguo Li [1,2] and Chandra S. Verma [1,3,4,*]

1 Bioinformatics Institute, Agency for Science, Technology and Research (A*STAR), 30 Biopolis Street, #07-01 Matrix, 138671, Singapore; pietroa@bii.a-star.edu.sg (P.G.A.A.); nguyenbinhchem@gmail.com (T.B.N.); lijg@bii.a-star.edu.sg (J.L.)

2 Singapore Eye Research Institute, Singapore 169856, Singapore

3 School of Biological Sciences, Nanyang Technological University, 60 Nanyang Drive, Singapore 637551, Singapore

4 Department of Biological Sciences, National University of Singapore, 14 Science Drive 4, Singapore 117543, Singapore

* Correspondence: raghavk@bii.a-star.edu.sg (S.K.); chandra@bii.a-star.edu.sg (C.S.V.); Tel: +65-6478-8353 (S.K.); Fax: +65-6478-9048 (S.K.); Tel: +65-6478-8273 (C.S.V.); Fax: +65-6478-9048 (C.S.V.)

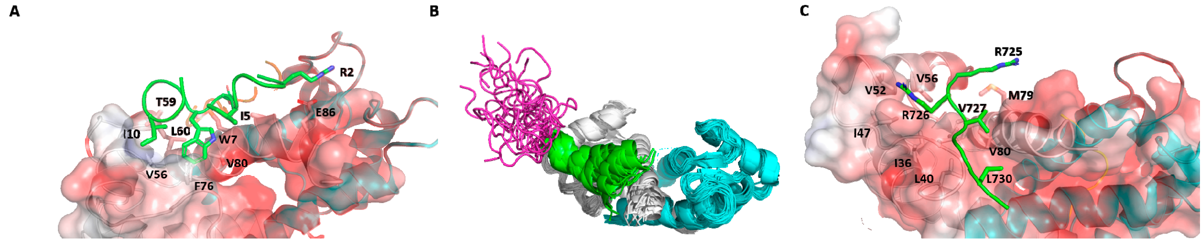

Figure S1: Structure of S100B(ββ) – peptide complexes. Structures of S100B(ββ) - (A) TRTK12_e, (C) RSK_PEP2, (E) RSK_L_e complexes are shown. The S100B(ββ) protein dimer is shown as electrostatic surface (red to blue colours represent electrostatic potentials ranging from -5 to +5 kcal/mol) with the two monomers coloured separately (grey, cyan). The bound peptide is shown as cartoon (green) and peptide – protein interacting residues and h-bond interactions are highlighted in sticks and dashed lines respectively. (B) NMR ensemble of S100B(ββ) – NDR peptide complexes, with the S100B(ββ) protein dimer coloured separately (grey, cyan) and the bound peptide is shown as cartoon with the alpha helix (green) and flexible region (magenta).

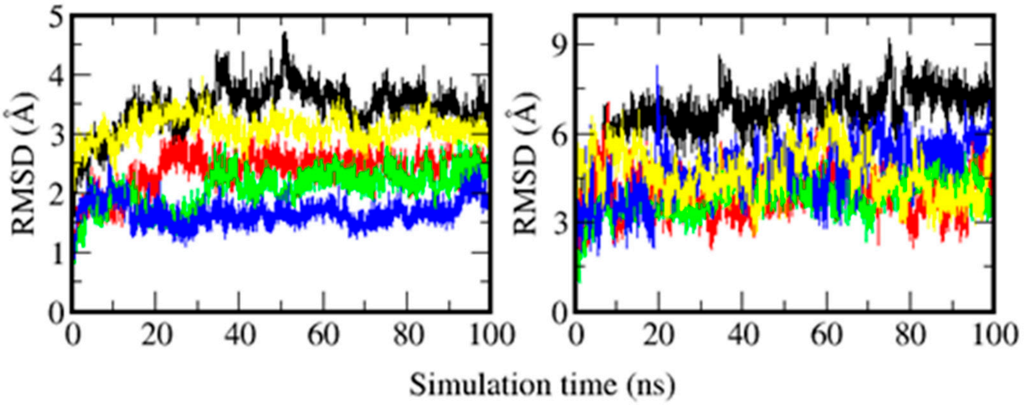

Figure S2: Root mean square deviation (RMSD) of the conformations (left) S100B(ββ) (right) and the bound peptides (red: NDR, green: RAGE, blue: RSK_PEP1, orange: RSK_PEP2, magenta: TRTK12) sampled during MD simulations.

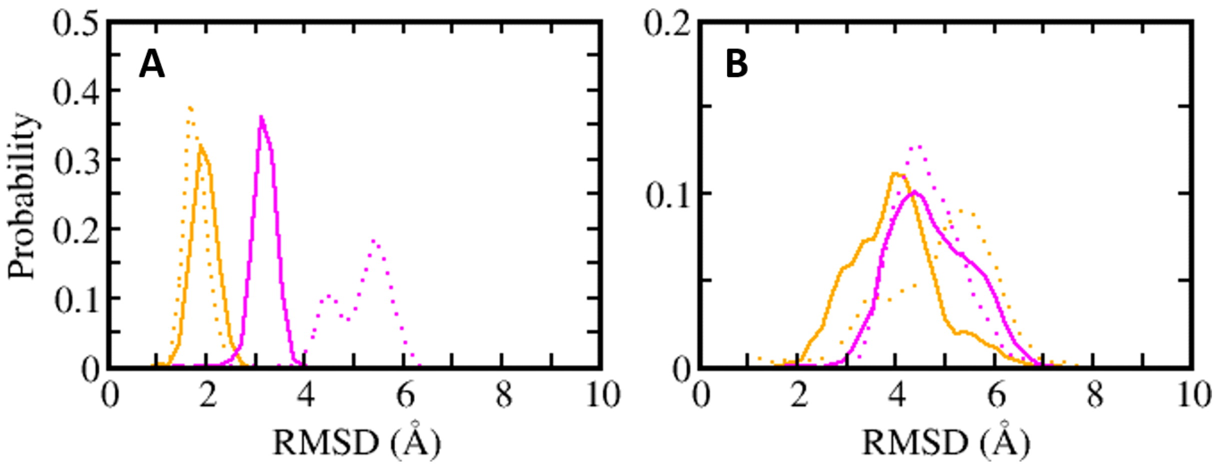

Figure S3: Distribution of Root mean square deviation (RMSD) of (A) S100B(ββ) (B) and the bound peptides in alpha helix (continuous line) and extended conformation (dotted line) (orange: RSK_PEP2, magenta: TRTK12).

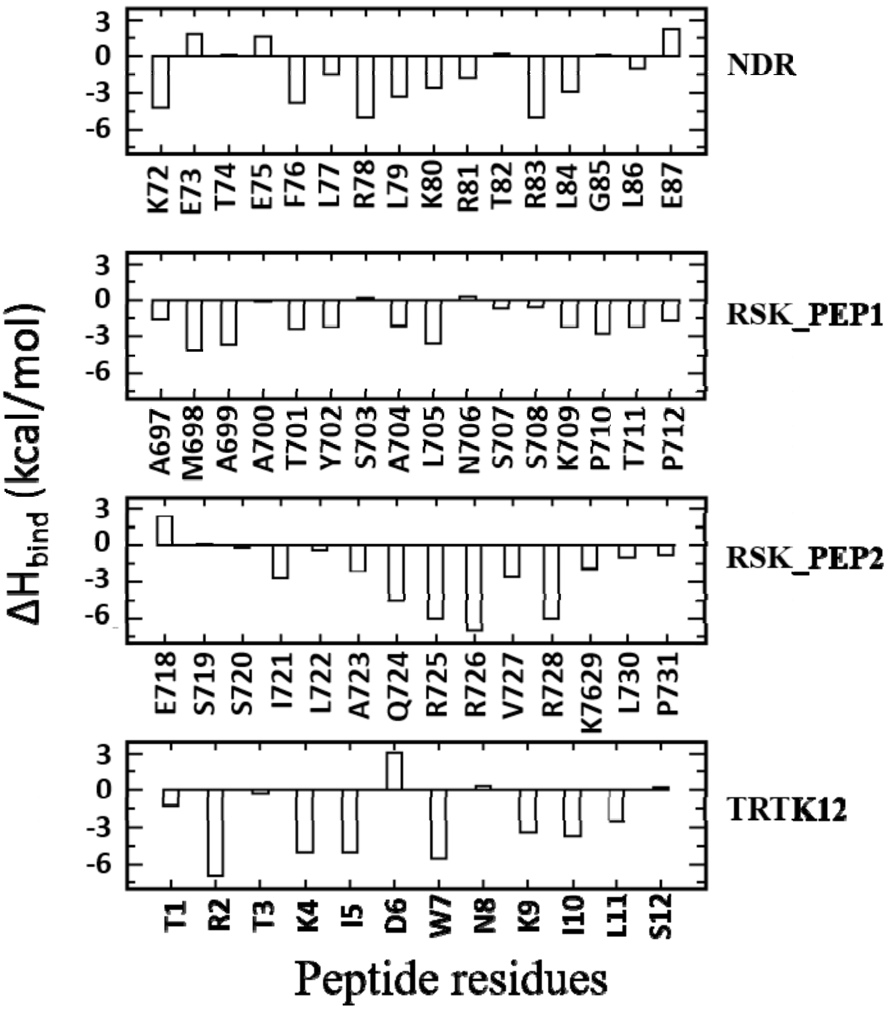

Figure S4: Per residue decomposition energetic analysis of the MD simulations of the S100B(ββ)– peptide complexes. Energetic contribution of each peptide residue to the binding energies of the S100B(ββ)– peptide complexes calculated with the MMGBSA using the conformations sampled during MD simulations.

Peptide		72	73	74	75	76	77	78	79	80	81	82	83	84	85	86	87		% helicity
NDR_WT	Ac-	K	E	T	E	F	L	R	L	K	R	T	R	L	G	L	E	NH2	46
NDR_SPEP1	Ac-	K	E	R8	E	F	L	R	L	K	S5	T	R	L	G	L	E	NH2	67
NDR_SPEP1_MUT2	Ac-	K	E	R8	E	*W*	L	R	*M*	K	S5	T	R	*F*	G	L	E	NH2	59
NDR_SPEP1_MUT5	Ac-	K	*L*	R8	E	*W*	L	R	*M*	K	S5	T	R	*F*	G	L	E	NH2	68
NDR_SPEP5	Ac-	K	E	T	E	F	L	R	L	K	R	S5	R	L	R5	L	E	NH2	55
NDR_SPEP5_MUT2	Ac-	K	E	T	E	*W*	L	R	*M*	K	R	S5	R	*F*	R5	L	E	NH2	57
NDR_SPEP5_MUT5	Ac-	K	*L*	T	E	*W*	L	R	*M*	K	R	S5	R	*F*	R5	L	E	NH2	49
NDRS_PEP7	Ac-	K	E	T	E	F	L	R	L	K	R	T	R	S5	G	L	R5	NH2	69
NDRS_PEP7_MUT2	Ac-	K	E	T	E	*W*	L	R	*M*	K	R	T	R	S5	G	L	R5	NH2	48
NDRS_PEP7_MUT5	Ac-	K	*L*	T	E	*W*	L	R	*M*	K	R	T	R	S5	G	L	R5	NH2	52

(Sequence)

Peptide		697	698	699	700	701	702	703	704	705	706	707	708	709	710	711	712		% helicity
RSK_PEP1_WT	Ac-	A	M	A	A	T	Y	S	A	L	N	S	S	K	P	T	P	NH2	34
RSK_PEP1_SPEP1	Ac-	A	M	A	A	T	S5	S	A	L	S5	S	S	K	P	T	P	NH2	36
RSK_PEP1_SPEP1_MUT3	Ac-	A	M	A	*W*	*L*	S5	S	A	*M*	S5	S	S	K	P	T	P	NH2	31
RSK_PEP1_SPEP1_MUT6	Ac-	A	*L*	*Q*	*W*	*L*	S5	S	A	*M*	S5	S	*T*	K	P	T	P	NH2	39
RSK_PEP1_SPEP4	Ac-	A	M	A	A	T	Y	S5	A	L	R5	S	S	S5	P	T	P	NH2	37
RSK_PEP1_SPEP4_MUT3	Ac-	A	M	A	*W*	*L*	Y	S5	A	*M*	R5	S	S	S5	P	T	P	NH2	16
RSK_PEP1_SPEP4_MUT6	Ac-	A	*L*	*Q*	*W*	*L*	Y	S5	A	*M*	R5	S	*T*	S5	P	T	P	NH2	10
RSK_PEP1_SPEP6	Ac-	A	M	A	A	T	Y	R8	A	L	N	S	S	K	S5	T	P	NH2	54
RSK_PEP1_SPEP6_MUT3	Ac-	A	M	A	*W*	*L*	Y	R8	A	*M*	N	S	S	K	S5	T	P	NH2	41
RSK_PEP1_SPEP6_MUT6	Ac-	A	*L*	*Q*	*W*	*L*	Y	R8	A	*M*	N	S	*T*	K	S5	T	P	NH2	42
RSK_PEP1_SPEP10	Ac-	A	M	A	A	T	Y	S	A	L	S5	S	S	K	S5	T	P	NH2	59
RSK_PEP1_SPEP10_MUT3	Ac-	A	M	A	*W*	*L*	Y	S	A	*M*	S5	S	S	K	S5	T	P	NH2	54
RSK_PEP1_SPEP10_MUT6	Ac-	A	*L*	*Q*	*W*	*L*	Y	S	A	*M*	S5	S	*T*	K	S5	T	P	NH2	56
RSK_PEP1_SPEP11	Ac-	A	M	A	A	T	Y	S	A	L	N	S5	S	K	R5	T	P	NH2	22
RSK_PEP1_SPEP11_MUT3	Ac-	A	M	A	*W*	*L*	Y	S	A	*M*	N	S5	S	K	R5	T	P	NH2	41
RSK_PEP1_SPEP11_MUT6	Ac-	A	*L*	*Q*	*W*	*L*	Y	S	A	*M*	N	S5	*T*	K	R5	T	P	NH2	43

(Sequence)

Peptide		718	719	720	721	722	723	724	725	726	727	728	729	730	731		% helicity
RSK_PEP2_WT	Ac-	E	S	S	I	L	A	Q	R	R	V	R	K	L	P	NH2	52
RSK_PEP2_SPEP1	Ac-	S5	S	S	R5	L	A	Q	R	R	V	R	K	L	P	NH2	31
RSK_PEP2_SPEP1_MUT	Ac-	S5	*T*	*L*	R5	L	*F*	*T*	R	R	*L*	R	K	L	P	NH2	35
RSK_PEP2_SPEP2	Ac-	S5	S	S	I	S5	A	Q	R	R	V	R	K	L	P	NH2	34
RSK_PEP2_SPEP2_MUT	Ac-	S5	*T*	*L*	I	S5	*F*	*T*	R	R	*L*	R	K	L	P	NH2	49
RSK_PEP2_SPEP4	Ac-	E	S	S	S5	L	A	R5	R	R	V	R	K	L	P	NH2	34
RSK_PEP2_SPEP4_MUT	Ac-	E	*T*	*L*	S5	L	*F*	R5	R	R	*L*	R	K	L	P	NH2	19
RSK_PEP2_SPEP5	Ac-	E	S	S	I	S5	A	Q	R5	R	V	R	K	L	P	NH2	26
RSK_PEP2_SPEP5_MUT	Ac-	E	*T*	*L*	I	S5	*F*	*T*	R5	R	*L*	R	K	L	P	NH2	33

(Sequence)

Peptide		1	2	3	4	5	6	7	8	9	10	11	12		% helicity
TRTK12_WT	Ac-	T	R	T	K	I	D	W	N	K	I	L	S	NH2	20
TRTK12_SPEP1	Ac-	T	R	T	K	I	S5	W	N	R5	I	L	S	NH2	30
TRTK12_SPEP1_MUT1	Ac-	T	R	T	K	I	S5	W	N	R5	I	*M*	S	NH2	29
TRTK12_SPEP1_MUT3	Ac-	T	R	T	K	I	S5	W	N	R5	*L*	*M*	*L*	NH2	20
TRTK12_SPEP2	Ac-	T	R	T	K	I	D	W	S5	K	I	L	S5	NH2	34
TRTK12_SPEP2_MUT1	Ac-	T	R	T	K	I	D	W	S5	K	I	*M*	S5	NH2	33
TRTK12_SPEP2_MUT2	Ac-	T	R	T	K	I	D	W	S5	K	*L*	*M*	S5	NH2	31
TRTK12_SPEP3	Ac-	T	R	T	K	I	D	W	N	S5	I	L	R5	NH2	28
TRTK12_SPEP3_MUT1	Ac-	T	R	T	K	I	D	W	N	S5	I	*M*	R5	NH2	17
TRTK12_SPEP3_MUT2	Ac-	T	R	T	K	I	D	W	N	S5	*L*	*M*	R5	NH2	28

Figure S5: Sequences of the designed stapled mutant peptides are shown here. Residues that are linked through all hydrocarbon linkers i,i+3, i,i+4 and i,i+7 are highlighted in red and the mutations are in italics. The helicity (percentage) of peptides when bound to S100B(ββ) are shown.
